# Estimation of Soil Characteristics from Multispectral Sentinel-3 Imagery and DEM Derivatives Using Machine Learning

**DOI:** 10.3390/s23187876

**Published:** 2023-09-14

**Authors:** Flavio Piccoli, Mirko Paolo Barbato, Marco Peracchi, Paolo Napoletano

**Affiliations:** 1 Department of Informatics, Systems and Communications, Università degli Studi di Milano-Bicocca, 20126 Milano, Italy; m.barbato2@campus.unimib.it (M.P.B.); m.peracchi1@campus.unimib.it (M.P.); paolo.napoletano@unimib.it (P.N.); 2 Istituto Nazionale di Fisica Nucleare, Sezione di Milano Bicocca, Piazza della Scienza 3, 20126 Milano, Italy

**Keywords:** digital soil mapping, machine learning, multispectral sensing, Sentinel-3, digital elevation model

## Abstract

In this paper, different machine learning methodologies have been evaluated for the estimation of the multiple soil characteristics of a continental-wide area corresponding to the European region, using multispectral Sentinel-3 satellite imagery and digital elevation model (DEM) derivatives. The results confirm the importance of multispectral imagery in the estimation of soil properties and specifically show that the use of DEM derivatives improves the quality of the estimates, in terms of $R^{2}$, by about 19% on average. In particular, the estimation of soil texture increases by about 43%, and that of cation exchange capacity (CEC) by about 65%. The importance of each input source (multispectral and DEM) in predicting the soil properties using machine learning has been traced back. It has been found that, overall, the use of multispectral features is more important than the use of DEM derivatives with a ration, on average, of 60% versus 40%.

## 1. Introduction

Monitoring soil properties is a fundamental aspect of precision agriculture, offering improved resource management [1], enhanced risk assessment and effective land erosion monitoring [2]. Furthermore, soil has the potential for carbon sequestration, which could prove to be a formidable tool in combating climate change in the years ahead [3].

The primary method for the characterization of soil involves manually collecting soil samples, drying them and subsequently performing chemical analyses in a laboratory setting [4]. However, the manual collection of soil samples, along with their corresponding physicochemical characterization, is a time-consuming process that lacks scalability for extensive areas [5]. Different soil properties interact with electromagnetic radiation in diverse ways. As electromagnetic waves strike the Earth’s surface, they can be absorbed, transmitted or reflected. The reflection and absorption patterns at different wavelengths provide insights into the composition, structure and properties of the observed materials [6]. More recently, hyperspectral and multispectral soil characterization has emerged as a highly valuable tool for the estimation of soil properties without the need for chemical analyses of the soil samples [7,8]. Multispectral and hyperspectral remote sensing harness data from multiple narrow and contiguous bands across the electromagnetic spectrum, with each band corresponding to a specific wavelength range. This technology has proven to be immensely valuable for soil characterization due to its ability to detect and analyze various soil properties [6,9].

Remote sensing involves the use of sensors mounted on drones, aircraft, or satellites for observing and monitoring the Earth from a distance. When coupled with Machine Learning, this technology can also be applied to soil characterization, expediting and scaling up the soil characterization process [10]. The integration of these technologies facilitates accurate and high-resolution soil mapping, empowering land managers, farmers and policymakers with vital information for sustainable land use planning, precision agriculture and environmental conservation initiatives [11]. There are several research papers on the use of machine learning for soil parameter estimation from spectral images. Ladoni et al. [12] use partial least square regression to estimate the soil organic carbon (SOC) from hyperspectral images. Forkuor et al. [13] estimate the sand, silt, clay, CEC, SOC and nitrogen (N) on West African terrain starting from RapidEye and Landsat images, through the use of random forests (RF), support vector machines (SVM) and stochastic gradient boosting (SGB). Similarly, Safanelli et al. [14] estimate the clay, sand, SOC, calcium carbonates (CaCO_3_), CEC and pH present in water using the gradient boosting regression algorithm. Zhou et al. [15] use RF, SGB and SVM to predict the SOC and the C:N ratio from multispectral data for Switzerland [16]. Hu et al. [17] estimate the soil salinity with RF using hyperspectral and multispectral data. Guo et al. [18] perform the SOC prediction using vis-NIR (visible near-infrared) technology. Two approaches are compared: a direct method, which estimates directly the SOC from spectral information, and an indirect method, which in the first place estimates the soil organic matter (SOM) and the soil bulk density (SBD), and then computes the SOC value on the basis of the estimated variables. Meng et al. [19] estimate the SOC from hyperspectral images obtained from the Gaofen-5 (GF-5) satellite. The proposed multiscale approach uses the three bands having the highest correlation with the SOC as features and predicts the target variable with RF [20], SVM [21] and backpropagation neural networks. Chambers et al. [22] created two datasets for soil nutrient prediction (P, K and M) and then investigated the use of several machine learning techniques for the prediction. The two datasets are, respectively, called global and local datasets, as the former covers several locations in Slovenia while the latter corresponds to a local farm. Both datasets are augmented using subsampling, reaching a total of 350 and 56 samples, respectively. Data acquisition of the spectra was performed within the UV-VIS (ultra-violet visible) range, specifically between 200 nm and 11,000 nm. Li et al. [23] performed a prediction of soil properties (OC, N, and Clay) starting from vis-NIR signals. The investigation was conducted on two datasets: a small dataset collected by the authors and the LUCAS (Land Use and Coverage Area frame Survey) dataset [4]. The prediction is achieved through a multi-branch neural network that evaluates both the signal as-is and the corresponding 2D spectrum map, obtained through the use of the Fourier transform. The latter treats the vis-NIR as a temporal signal. Three different preprocessing methods are investigated: the Savitzky–Golay (S-G) smoothing algorithm [24,25], multivariate scattering correction (MSC) [26,27] and centralization methods.

Among the aforementioned research papers, the work authored by Zhou et al. [15] stands out as one of the most pertinent contributions. This study presents a comprehensive comparison of diverse satellite sensors (including Landsat-8, Sentinel-2 and Sentinel-3), each coupled with varying spatial and temporal resolutions, in an effort to predict the organic carbon content and C:N ratio in Switzerland. The outcomes of this analysis show that the prediction models based on Landsat-8 and Sentinel-2 yielded the most favorable and least favorable results, respectively, in terms of error in the organic carbon estimations. It is worth noting, however, that this investigation also highlighted the potential inherent in models based in Sentinel-3 data. Despite its coarser resolution in comparison to Landsat-8 and Sentinel-2 (300 m versus 30 m and 10–60 m), models utilizing Sentinel-3 exhibited competitive or even superior accuracy. Remarkably, Sentinel-3’s advantage lies in its broader spectral coverage, offering 21 bands as opposed to the 7 of Landsat-8 and the 13 of Sentinel-2. This expanded spectral range holds the promise of enhancing the estimations of soil parameters. Moreover, it is important to emphasize that Sentinel-3, while characterized by reduced spatial resolution, represents a relatively novel sensor that remains largely unexplored for machine learning-based soil parameter estimation, as highlighted by Odebiri et al. [28]. One limitation of this study [15] is the extent of the area under investigation, which is limited to the Swiss territory. In fact, the amount of data used in the experiments is limited, making it difficult to use data-hungry methodologies such as machine learning. The size of the area under investigation is very important in terms of demonstrating the generalization capabilities of soil estimation methods on regions not considered during the training phase. In fact, soil properties vary significantly across different regions due to their unique physicochemical properties, resulting from factors such as climate, topography and time [29]. Moreover, terrain features, including slope, aspect and elevation, along with environmental elements like water availability and vegetation, play a crucial role in influencing spectral transmission, which is essential in our application [30].

Starting from these aspects, we present a study that shows the effectiveness of soil parameter estimation models over a larger geographical area than Switzerland. In more detail, in this paper, we evaluate different machine learning methodologies for the estimation of the multiple soil characteristics of a continent-wide area corresponding to the European region using multispectral Sentinel-3 satellite imagery [31] and DEM [32] derivatives. The soil characteristics’ ground-truth is obtained from the LUCAS library, which is the largest collection of physicochemical soil properties and corresponding spectral reflections acquired in the laboratory. The LUCAS library includes about 20,000 samples taken from specific geographical locations across the entire European region [4]. We associated with each geographical location of the LUCAS dataset the corresponding Sentinel-3 multispectral signature and DEM derivative, thus obtaining a large remote sensing dataset to be used for the estimation of multiple soil characteristics. Our study area includes the entire European region, comprising an extensive collection of soil samples with remarkable diversity and heterogeneity. The analysis presented in this paper provides insights into the potential of machine learning techniques to generalize over a vast geographical area. Nevertheless, given the substantial variations in soil properties across different regions, as mentioned earlier, the validity of these findings for areas beyond Europe should be empirically verified.

The main contributions of this paper are as follows:

- We created a multisource remote sensing dataset of the European region by merging multispectral images from Sentinel-3 and DEM derivatives from the European Copernicus mission and the corresponding LUCAS samples;
- We benchmarked several machine learning methods for the estimation of the soil characteristics using multispectral signals, DEM derivatives and a combination of them;
- We proposed methods based on an artificial neural network (ANN) capable of predicting all the soil characteristics at the same time;
- We analyzed the importance of each input source (multispectral and DEM) in predicting the soil properties.

Section 2 presents the machine learning-based methods for the soil characteristic estimation used in this paper. Afterward, the procedure used to collect the data from the different sources in order to create a real-case library for the spaceborne estimation of soil properties is described in Section 3. Finally, in Section 4 the results, together with a comparison with state-of-the-art methods, are presented and discussed.

## 2. Methods

Machine learning has revolutionized various scientific disciplines by enabling computers to learn patterns from data and make precise predictions. Several methodologies have been presented in the state of the art, such as ANNs, gradient boosting (GB), random forest (RF), support vector regressor (SVR), etc. Among these methods, ANNs have emerged as a powerful class of data-driven algorithms inspired by the biological neural networks of the human brain. These methods have gained significant popularity due to their ability to deal with complex and high-dimensional data, making them well suited for a wide range of applications, such as classification and semantic segmentation. In this study, we leverage the potential of ANNs in combination with multispectral Sentinel-3 satellite imagery and DEM derivatives to estimate multiple soil characteristics over a continent-wide area corresponding to the European region. ANNs optimize their performance through a data-driven training procedure that minimizes a loss function by employing interconnected nodes and weighted connections to learn from the data. The use of data-driven ANNs in our research provides an efficient method for the improvement of the precision of soil property estimation, and their application in conjunction with multispectral features and DEM derivatives demonstrates their crucial role in advancing soil characterization using machine learning techniques.

A machine learning method for the estimation of a soil parameter *s* learns a mapping function f(u;Θ), with parameters Θ, between a given input u and a target soil parameter *s*. The input can be either a multispectral signature m alone or a combination of multispectral and DEM features d. This method can be extended for the estimation of multiple soil parameters $s=\{s_{1},\cdots ,s_{P}\}$, with *P* being the number of parameters to be estimated. At the inference time, given the input u, the mapping function f(u;Θ) outputs the estimation $\hat{s}$ of a given soil property. In the case of multi-variable estimation, the mapping function outputs the estimation of all the variables at the same time $\hat{s}=\left\{{\hat{s}}_{1},\cdots ,{\hat{s}}_{P}\right\}$. The goodness of the predictions can be evaluated by comparing *s* with $\hat{s}$ in the case of single-variable estimation, and by comparing s with $\hat{s}$ in the case of multiple-variable estimation. The metrics used for the evaluation are the coefficient of determination $R^{2}$, root mean square error (RMSE) and mean absolute error (MAE).

In this paper, we have considered the following four state-of-the-art methods [15]: ANNs, GB, RF and support vector regressor (SVR). For all these methods, we evaluated the use of two different inputs, namely (i) multispectral information (m); and (ii) a combination of multispectral information (m) and features extracted from the DEM, which we refer to as DEM derivatives (m,d). While for the GB, RF, and SVR, we adopted the canonical implementation available from the Python Scikit-Learn library (https://scikit-learn.org/stable/, accessed on 1 August 2023), in the case of the ANNs, we specially engineered a network architecture for the soil parameter estimation task. GB, SVR, and RF follow the same configuration used by Zhou et al. [15]. For all of them, the parameters were optimized using the grid search algorithm.

The GB algorithm uses regression trees and gradient optimization as a procedure for the minimization of the loss function. The algorithm consists of training a set of regression trees in sequence. At each step, the residual error of the previous tree is used as a label for the current tree. After the prediction of all the trees, gradient optimization is used to change the weights of each tree, minimizing the loss function. The loss function considered in our experiments is the mean squared error (MSE) (Equation (1)).

(1) $$MSE=\frac{1}{N}\sum_{i=1}^{N}{\left(x_{i}-y_{i}\right)}^{2}$$

where $x_{i}$ and $y_{i}$ are the corresponding pair of the input and output, and *N* is the number of samples.

SVR, instead, is a machine-learning algorithm that allows for a definition of a tolerance on the accepted error of the model. Based on a kernel function, it allows for the identification of the best hyperplane to fit the data. The kernel function used in the experiments is the radial basis function (RBF) (Equation (2)), due to its performance in soil mapping [33].

(2) $$k\left(x_{i},x_{j}\right)=exp\left(-\sigma ∥x_{i}+x_{j}{∥}^{2}\right)$$

where *k* is the kernel function, $x_{i}$ and $x_{j}$ are the input vectors, and σ represents the width of the RBF, thus regulating the relationship between input and output.

RF uses multiple decision trees to predict the output. Each tree makes its own prediction, and the final values are obtained by merging them together. RF also uses a bootstrap technique to generalize the training and avoid overfitting [34]. Basically, every time a prediction is made during the training, a set of N samples from the dataset is chosen and used for the training step. The remaining samples are used to evaluate the value of the loss function, which is then used during the optimization of the model. In this case also, the loss function chosen is the MSE (Equation (1)). The number of trees and the number of samples for the bootstrap procedure were optimized with a grid search algorithm, as was the case for the other tested methods.

The ANNs were implemented using the PyTorch Library (https://pytorch.org/, accessed on 1 August 2023). The architecture and hyperparameters of the model were optimized using a grid search method. The optimized architecture of the ANNs is described in Table 1. It is composed of five linear layers, including input and output layers. The three hidden layers are separated in turn by Tahnhshrink and Hardswish (h-swish) activation functions [35], which were selected during the optimization of the architecture. The input and output layers of the ANNs were parameterized so that they can be adapted to different types of inputs ((m) and (m,d)) and outputs. In fact, we also investigated the ability of the ANN to predict multiple soil properties at the same time, thus having two kinds of outputs: single and multi. To distinguish between the ANN for single-variable estimation and the ANN for multiple-variable estimation, we use the following labels: ANN Single and ANN Multi. The loss function used to train the ANN models is the MSE (Equation (1)). Furthermore, the hyperparameter optimization selected the Adam optimizer with a learning rate of $10^{-2}$ as the best-performing configuration.

### 2.1. Explainability Investigation

Data-driven methods automatically learn the importance of an input feature in predicting a given output. The more closely correlated the input feature is with the output, the more frequently it will be considered during the learning process. However, this correlation cannot be observed directly and requires an appropriate strategy that depends on the machine learning method adopted for the prediction [36].

In this paper, we have focused on the RF method for the analysis of the feature importance, with the aim of understanding which bands of the multispectral signal and which DEM derivatives contribute most significantly to a given soil variable estimation. Due to their remarkable effectiveness and interpretability, RFs have acquired great popularity in the literature. This unique combination establishes them as a potent tool not only for accurate forecasting but also for the facilitation of a comprehensive explanation of outcomes.

Two strategies exist in the state of the art: the mean decrease in impurity (MDI) and the feature permutation (FP) [20,37]. The former counts the number of times a feature is used to split a tree node, weighted by the number of samples it splits. In the version used in this investigation, the decrease in node impurity is also weighted by the probability of reaching that node. The latter, instead, measures the increase in the prediction error of the model after the values of the features are permuted, breaking the relationship between the features and the true outcome. The impurity-based feature importance cannot scale up well on high-cardinality input features. Therefore, we adopted the FP technique.

## 3. Materials

One of the contributions of this work is the creation of a large library of soil properties, spaceborne multispectral data and digital elevation data corresponding to *N* geo-referenced land points. The entire library is defined as L=(S,M,D), with $S={\left\{s_{i}\right\}}_{n=1}^{N}$, $M={\left\{m\right\}}_{n=1}^{N}$ and $D={\left\{d\right\}}_{n=1}^{N}$ being the soil properties, multispectral and digital elevation model information, respectively.

For each land point *n*, $s_{n}$, $m_{n}$ and $d_{n}$ are defined as follows:

- $s_{n}=\left\{s_{1}^{n},\cdots ,s_{P}^{n}\right\}$ are the P=12 soil properties;
- $m_{n}=\left\{b_{1}^{n},\cdots ,b_{L}^{n}\right\}$ are the L=21 bands of the spaceborne multispectral data;
- $d_{n}=\left\{d_{1}^{n},\cdots ,d_{K}^{n}\right\}$ are the K=8 features extracted from the digital elevation model information.

In the following section, we present, first, how we collected and processed the multispectral data, and secondly, how we selected the soil properties and created an association between the properties and the spectra. Finally, we describe how we collected the digital elevation data and extracted the features to be associated with the soil properties.

### 3.1. Multispectral Data

We gathered data from the Sentinel-3 satellite mission in order to cover the entire European continent. Sentinel-3 is a multi-instrument mission to measure the sea surface topography, sea and land surface temperature and ocean and land color with a high level of accuracy and reliability. The mission is composed of two satellite platforms: Sentinel-3A, launched on 16 February 2016, and Sentinel-3B, launched on 25 April 2018.

Both satellites are equipped with several different sensing instruments and are collocated in the low Earth orbit. Among all the instruments, the Ocean and Land Colour Instrument (OLCI) is the most interesting for the purpose of this paper. OLCI has a spatial resolution of 300 m and is capable of measuring 21 spectral bands, from 400 nm to 1020 nm. It is capable of producing images with a swathe of 1270 km, and is not centered at the nadir, but is tilted 12.6° westwards to mitigate the negative impact of sun glint contamination. OLCI images are processed and distributed through the Copernicus Open Access Hub [38].

For this work, we used images preprocessed with level 1, which includes top-of-atmosphere (TOA) radiometric measurements, radiometrically corrected, calibrated and spectrally characterized. These images are also quality-controlled and georeferenced (latitude, longitude and altitude). The images cover an area of 1200 km${}^{2}$ and have a resolution of 300 m. All the images are encapsulated in a Network Common Data Form 4 (NetCDF 4) format [39] and processed through QGIS Desktop (version 3.20.2) software [40].

Images of level 1 were downloaded from the Copernicus Open Access Hub, using the Semi-Automatic Classification Plugin for QGIS (version 7.8.35) [41]. This tool allowed the downloading of images from the hub and computing preprocessing operations specific for Sentinel-3, creating 21 images corresponding to the 21 spectral bands. Atmospheric correction DOS1 (dark object subtraction) was applied [42]. This methodology is one of the most common techniques adopted for such purposes: water, forests and shadows are considered dark objects when their values of reflectance are close to zero. Dark objects are detected automatically when the pixel reflectance value is less than or equal to 1.0%. The assumption is that some pixels within the image receive 0% of solar radiation and the values of radiance corresponding to these pixels registered by the satellite correspond to atmospheric dispersion.

**Figure 1:**
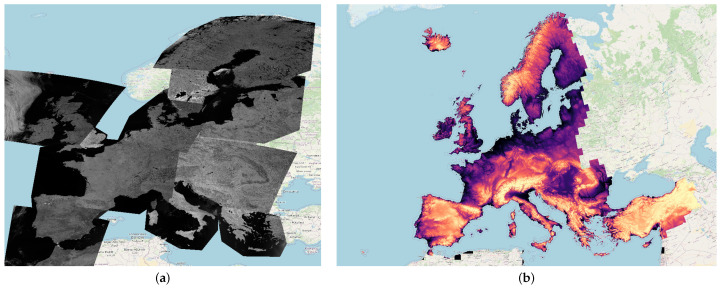
Representation of the data used in these experiments. (**a**) shows multispectral images collected by Sentinel-3 and properly merged to cover the entire European continent. For the sake of visualization, (**a**) represents the average over all the bands, normalized between 0 and 1. (**b**) represents the digital elevation model acquired from the Copernicus Land Monitoring Service. Greenish colours represent low-elevation values (approx. −214 m), while reddish colours represent high-elevation values (approx. 5105 m).

A total of 14 images were downloaded, spanning five years, from 2016 to 2021, with an acquisition time between the summer period, from May to September. Similarly to the practice adopted by Zhou et al. [15], all images were chosen with a cloud coverage inferior to 10% of the acquisition. The images were loaded into the QGIS software as single raster layers, with the same datum, EPSG:4326 World Geodetic System 1984, considered for each. Figure 1a shows all the multispectral images collected by Sentinel-3 and properly merged to cover the entire European continent. It should be noted that, for the sake of visualization, all the downloaded images are visualized by showing their spectral average, and thus, the resulting patchwork effect is only a visual artifact.

### 3.2. Soil Data

**Figure 2:**
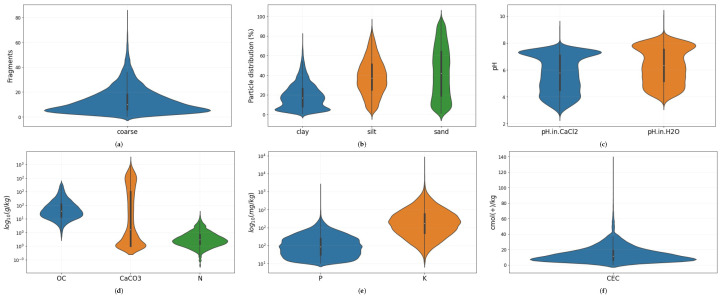
Violin plots of the soil properties considered in this work. (**a**) Coarse; (**b**) Clay, Silt and Sand; (**c**) pH in $CaCl_{2}$ and $H_{2}O$; (**d**) Organic Carbon (OC), Calcium Carbonate (CaCO_3_) and Nitrogen (N); (**e**) Phosphorous (P) and Potassium (K); (**f**) Cation exchange capacity (CEC). For the sake of visualization, the variables P, K and CEC are shown in a logarithmic scale.

To connect soil properties with each multispectral signature, we included the target variables considered in the LUCAS library. LUCAS is a programme carried out by EUROSTAT (the European Statistical Office) that aims to organize harmonized surveys across all the states of the European Union over time [4]. The LUCAS library includes a total of approximately 20,000 samples, each of 0.5 kg of topsoil material. The topsoil sampling locations were selected to be representative of the European landscape features. The selection was based on a stratified random sampling that took into consideration the CORINE land cover 2000, the Shuttle Radar Topography Mission (SRTM) DEM and its derived slope, aspect and curvature [43]. The authors of the dataset decided to exclude areas above 1000 m from the survey due to the challenges associated with accessing and sampling these high-altitude locations. Finally, the LUCAS topsoil sample points exhibit a density of approximately 1 per 199 km${}^{2}$, which theoretically permits a grid cell size of approximately 14 km [43]. All the dried samples were analyzed for the percentage of coarse fragments, particle size distribution (% clay, silt, and sand content), pH (in $CaCl_{2}$ and $H_{2}O$), organic carbon (g/kg), carbonate content-CaCO${}_{3}$ (g/kg), phosphorous content (mg/kg), total nitrogen content (g/kg), extractable potassium content (mg/kg), CEC (cmol(+)/kg) and hyperspectral reflections, measured in a laboratory environment. A great portion of the data, namely 43% of all samples, was collected from croplands. Figure 2 shows violin plot distributions of all the 12 soil properties. Figure 2d,e are in the logarithmic scale. The association of each LUCAS sample to the spaceborne multispectral signatures of Sentinel-3 has been achieved through the GPS coordinates associated with each sample.

### 3.3. Digital Elevation Model Data

To take into account the geometrical distortions of the land in the estimation of the soil properties, we included the DEM, which is a representation of the terrain elevation [44]. The DEM was acquired from the Copernicus Land Monitoring Service, resampled to a resolution of 8 m, and then saved as a raster layer on QGIS. Moreover, the DEM was reprojected from the EPSG:4326-WGS 84 datum, in degrees, to another datum (EPSG:3035 ETRS89-extended/LAEA Europe), which is in meters. Figure 1b shows the maps obtained, which were later sampled with the points from the LUCAS dataset.

Afterward, the raster layers corresponding to the DEM information were processed using the SAGA GIS software (version 7.8.2) [45] to extract the following features: altitude, valley depth, slope, topographic wetness index (TWI), channel network base level (CNBL), vertical distance to channel network (VDCN), catchment slope and slope length.

The TWI is used to estimate where water is accumulated, the CNBL is the base level of groundwater and the VDCN is the vertical distance. The exact procedure for the feature extraction is described as follows. We call the *valley depth* function with the following parameters: *Tension Threshold* 1, *Maximum Iterations* 0, *Keep Ridge Level Above Surface* checked, *Ridge Detection Threshold* 4. We call the function *slope* [46] (*Unit* radians) and *slope length*. Then, we call the function to calculate the *SAGA Wetness Index*, catchment area and catchment slope [47] with the following parameters: *Suction* 10, *Type of Area* square root of the catchment area, *Type of Slope* catchment slope, *Minimum slope* 0, *Offset Slope* 0.1, *Slope Weighting* 1. The channel network was created through the catchment area as an initiation grid, setting the initiation type as “Greater than” and the “threshold” as 10 million, using the *channel network* function with the following parameters: *Min. Segment Length* 10. Finally, the function *vertical distance to channel network* was used to extract the VDCN and CNBL with the following parameters: *Tension Threshold* 1, *Maximum Iterations* 0, *Keep Base Level below Surface* checked.

### 3.4. Comparison with Existing Datasets

Table 2 recapitulates the main characteristics of the state-of-the-art soil parameter estimation experiments in comparison with our proposal. All of these estimate a single or small subset of soil characteristics. Of great importance is the constraint on the volume of data employed in the experiments, posing challenges for the utilization of data-hungry approaches like machine learning. Lastly, the geographical area under test is usually region- or state-wide, with none of the previous works evaluating larger areas, such as continent-wide areas. Our proposal is the only one that considers a large set of multiple variables at the same time. Most importantly, our proposal includes the largest area, covering a continent-wide area corresponding to the European region.

### 3.5. Data Split

All the experiments were carried out on the proposed dataset, which was divided into training, validation and test sets, using the rule 80%-10%-10%. We ensured that the quantiles for each variable were the same in all the sets in order to have the same data distribution in all the sets.

Each soil property $s_{i}^{n}$ was normalized using a robust scaler [48]. The use of this preprocessing allows for a mitigation of the scale effect and the effects of outliers. The robust scaler subtracts the mean and scale on the base of the interquartile range, leading to a more robust rule. Formally, it is defined as:

(3) $$x_{i}^{n}=\frac{s_{i}^{n}-median\left(s_{i}\right)}{IQR_{0.25-0.75}}$$

where $s_{i}^{n}$ is the *i*-th soil property at the original scale while $x_{i}^{n}$ is the same property preprocessed with the robust scaler. $IQR_{0.25-0.75}$ is the interquartile range starting from the 25% quartile to the 75% quartile. Furthermore, the input features (m and d) were normalized to the zero mean and unit standard deviation.

## 4. Results

In this section, we assess the performance of all the methods considered. We also present an analysis of the feature importance in order to highlight the role of each single feature in the soil parameter prediction.

### 4.1. Experiments

Table 3a–c show the results achieved by all the methods considered and measured with $R^{2}$, RMSE and MAE, respectively. Two groups of experiments are shown, depending on the input features: multispectral input (m) and multispectral input with DEM derivatives (m,d). For each group of experiments, we show the performance of the state-of-the-art and ANN methods. For each input, the underlined values highlight the best methods for each soil variable, while the bold values highlight the best methods on average.

**Figure 3:**
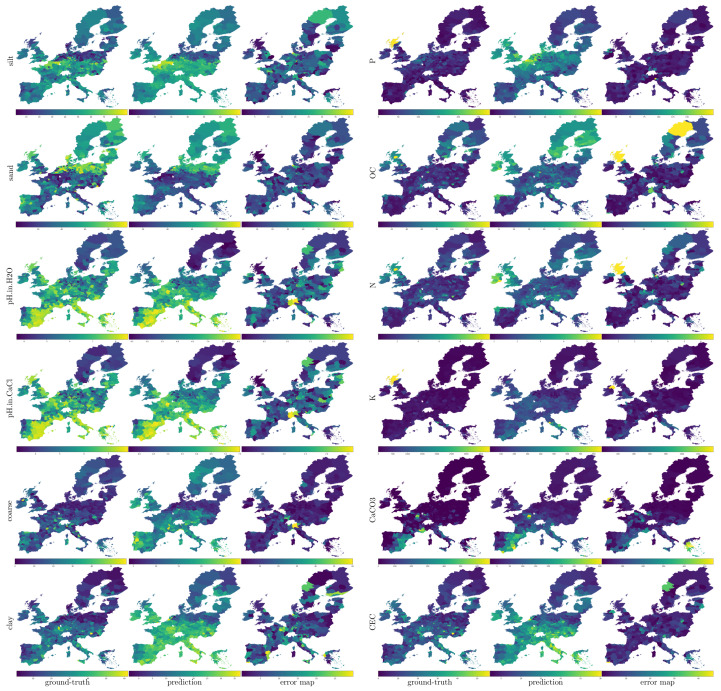
For a visual comparison of the results, we render through inverse distance weighting (IDW) interpolation the ground points relative to the ground-truth, the predictions, and the errors in terms of RMSE of each soil variable. The blue and yellow colors represent the minimum and the maximum values of each soil property, respectively. Colors relative to intermediate values are obtained through quantile color coding. It is worth noting that in most cases, the ground-truth (the first image of each triplet) and the prediction (the second image) are visually similar, indicating an accurate estimation of the soil properties.

ANN Single and ANN Multi are the best-performing methods in terms of $R^{2}$, whatever the input features are. ANN Single and ANN Multi, with the multispectral input (m), are the best-performing methods in terms of RMSE and MAE. In terms of RMSE, the best-performing methods when the inputs are the multispectral data and DEM derivatives (m,d) are ANN Single and RF. In terms of MAE, the best-performing method when the inputs are the multispectral data and DEM derivatives (m,d) is the SVR. To enable a visual comparison of the results, in Figure 3, we have rendered through inverse distance weighting (IDW) interpolation the ground-truth, predictions and errors in terms of RMSE for each soil variable. As it is possible to observe, in most cases, the maps corresponding to the ground-truth and the prediction of each soil variable are visually similar, indicating an accurate estimation. In agreement with the numerical results presented in Table 3, CaCO_3_, pH${}_{H_{2}O}$ and pH${}_{CaCl_{2}}$ are the ones that appear most similar. Interestingly, it is worth noting that the spatial distribution of the error is not homogeneous. In fact, an observation of the error maps of each triplet (last map) makes it clear that for each variable, there are zones where the estimation is less accurate. This could be due to several factors. First, although the sample collection is standardized, it is subject to human factors and errors. Secondly, there may be environmental factors that mitigate the observability of the phenomenon.

Instead, focusing on the $R^{2}$, (see Table 3a), it is possible to observe that some variables can be estimated more accurately than others. For instance, the methods for the estimation of pH$H_{2}O$ and pH${}_{CaCl_{2}}$ achieved an $R^{2}$ value higher than 0.5. On the contrary, the methods for the estimation of the variables *coarse*, *P* and *N* achieved an $R^{2}$ value lower than 0.20. Overall, the use of DEM derivatives permits the achievement of an increment of 19% and 15% in the case of ANN Single and Multi, respectively. In particular, the use of DEM derivatives improved the performance in the estimation of soil textures (silt, sand, coarse, and clay), in terms of $R^{2}$, on average by 43% and 30% in the case of ANN Single and ANN Multi, respectively. In the case of ANN Multi, the improvement is mitigated by the fact that this method predicts multiple variables at the same time. This result was expected since the soil properties are closely related to geological formations and landscape positions and, in particular, the soil textures are highly correlated to the parameters derived from the DEM [49]. Furthermore, the use of DEM significantly improved the estimation of the parameter CEC by about 65%, whichever method is employed. This behavior is due to the fact that the CEC is often correlated with the DEM because exchangeable cations can be mobilized and leached to lower landscape positions [50].

**Figure 4:**
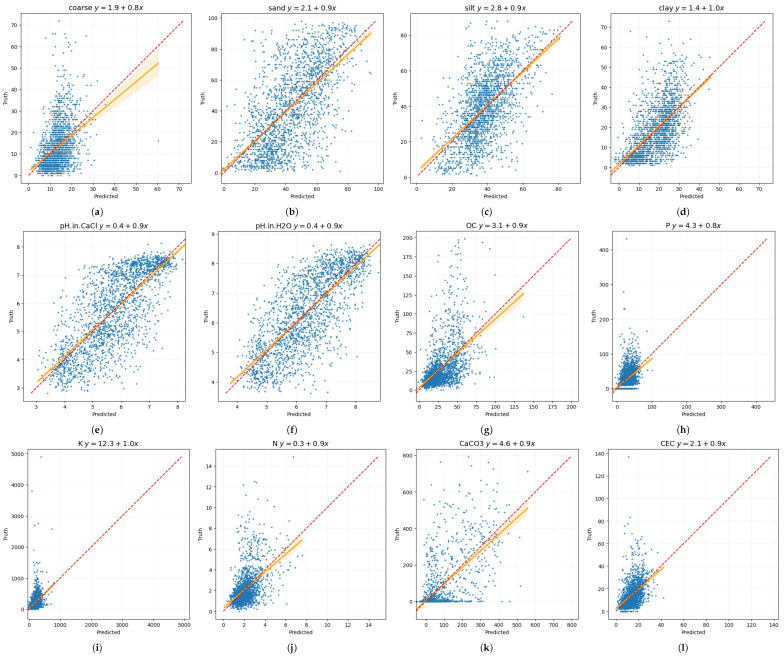
Scatter plots corresponding to the predictions of ANN Multi in relation to the soil parameters. The *x* axis refers to the predictions, while the *y* axis refers to the ground-truth values. For reference, each plot includes the perfect prediction line y=x (dashed red) and the trend line relative to the estimations (solid orange). Each subfigure represents the scatter plot of a given soil variable.

Figure 4 shows, for each soil variable, the scatter plots gathered by the best overall model (ANN Multi). Each plot shows the prediction vs. the ground-truth values, and ideally, each point should lie on the bisect of the graph. We also show the trend line (in orange) and provide its coefficients. As it is possible to observe, the trending lines are very close to the bisect, indicating a good quality of the results. Finally, to summarize the results achieved by each method and to show which combination of types is best, Table 4 reports, for each soil parameter and each metric (considering both (**m**) and (**m**,**d**)), the best technique for the estimation. For all the soil parameters, apart from K, the use of DEM in combination with multispectral imagery improves the estimation accuracy whichever evaluation metric is used. It is worth noting that in some cases, ANNs struggle to exploit the multimodality (**m**,**d**) with respect to other machine learning methods, which in turn adapt better to a diverse input. Nevertheless, ANNs on average outperform all the other methods evaluated, as confirmed in Table 3a,b.

### 4.2. Explainability Discussion

As described in Section 2.1, we also investigated the importance of an input feature in predicting a given output using a feature permutation approach. This investigation is fundamental to an understanding of the actual advantages of using the combination of spectral and DEM information to describe the property of the soil.

**Figure 5:**
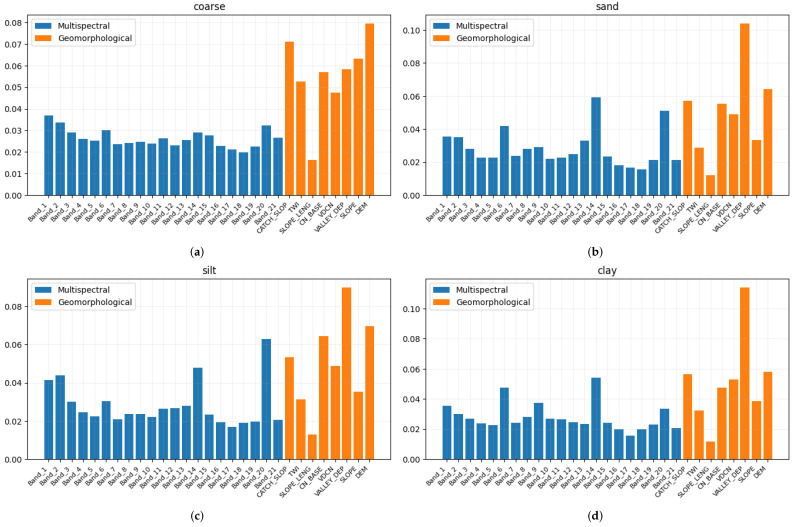

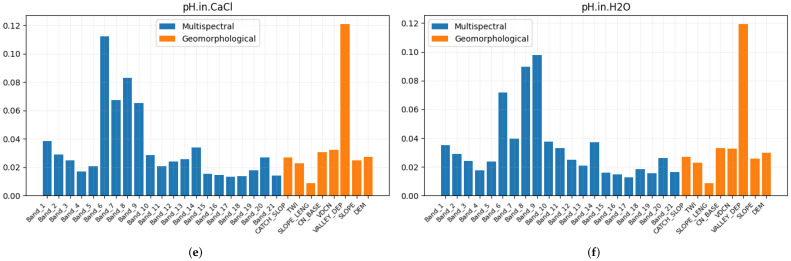
Feature importance of each soil property obtained using random forests. The blue bars represent the importance of the multispectral bands, while the orange bars are relative to the DEM-derivatives. (**a**) Coarse; (**b**) Sand; (**c**) Silt; (**d**) Clay; (**e**) pH in $CaCl_{2}$; (**f**) pH in $H_{2}O$.

**Figure 6:**
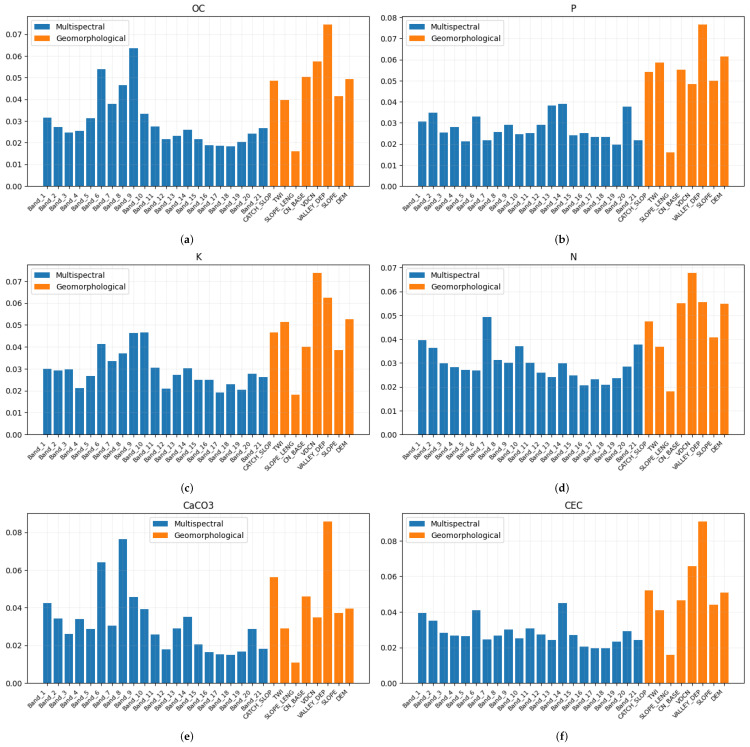
Feature importance of each soil property obtained using random forests. Blue bars represent the importance of the multispectral bands, while orange bars are relative to the DEM-derivatives. (**a**) Organic Carbon (OC); (**b**) Phosphorous (P); (**c**) Potassium (K); (**d**) Nitrogen (N); (**e**) Calcium Carbonate (CaCO_3_); (**f**) Cation exchange capacity (CEC).

Figure 5 and Figure 6 show the feature importance in the estimation of each soil variable. The blue bars represent the bands of the multispectral signal, while the orange bars represent the DEM derivatives. It is possible to observe that for some variables, such as *coarse* and *P*, all the bands have more or less the same significance, while with respect to other variables, there are spiking bands that heavily impact on the results, such as bands 8 and 9 in the estimation of pH in $H_{2}O$.

In the case of the geomorphological features, the band that most significantly impacted on the predictions is the *valley depth*. This is because the *valley depth* is a vital indicator of a depositional (sedimentary) environment [51]. On the contrary, the *slope length* does not provide a significant contribution to the prediction of the soil properties.

**Figure 7:**
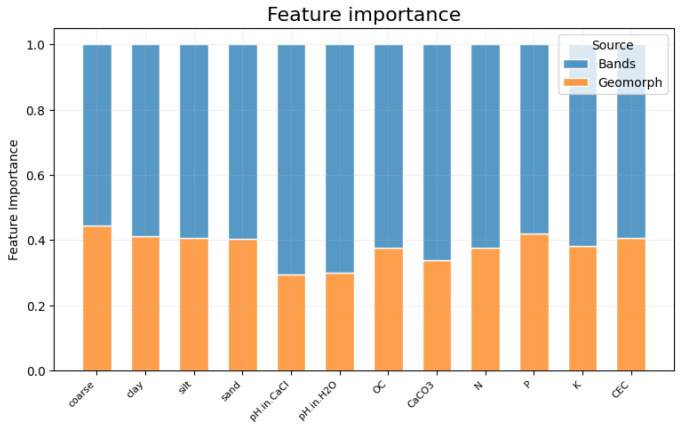
Aggregated feature importance of each soil variable. The contributions of the multispectral and geomorphological DEM derivatives are shown in blue and orange, respectively. It is worth noting that the importance of the multispectral variables is greater than that of the DEM derivatives.

Figure 7 shows the comparison between the two groups of features we considered (multispectral and DEM) in terms of their importance in the estimation of the soil variables. For a given variable, blue and orange bars represent the percentage of multispectral bands and DEM, respectively. Overall, the use of multispectral features is more important than the use of DEM derivatives with a ratio on average of 60% vs. 40%. In some cases, such as the estimation of pH${}_{CaCl}$ and pH${}_{H_{2}O}$, the use of DEM derivatives counts for about 30%, thus confirming that geomorphological features are less important in the estimation of pH levels.

## 5. Final Discussion and Conclusions

In this work, we have evaluated different machine learning-based methods for the estimation of the multiple soil characteristics of a continent-wide area corresponding to the European region from multispectral signal and DEM derivatives. The multispectral signals, DEM derivatives, and soil characteristics have been gathered respectively from the Sentinel-3 satellite, the European Copernicus mission and the LUCAS library, respectively. All the data collected were then geographically matched to create a uniform and multisource benchmark of 20,000 samples. On this dataset, we benchmarked several machine learning methods representing the state of the art for the estimation of soil characteristics. These methods were adapted to use multispectral signals, DEM derivatives and a combination of each of them. We also proposed an ANN-based method capable of predicting all the soil properties at the same time and included this in the investigation. Three metrics were used for the performance assessment: MAE, RMSE and $R^{2}$. Overall, neural networks showed the best performance in describing the data. Our experiments also demonstrated how the use of DEM derivatives improves the quality of the predictions in contrast with the use of the multispectral signal alone. The improvement in terms of $R^{2}$ increment is 19% on average, this being greatly appreciated in the prediction of soil textures where it reaches the 43%. Moreover, the correlation between DEM and CEC has allowed for a significant improvement of about 65%. Further analysis of the feature importance revealed a high impact of the multispectral bands 8 and 9 in the estimation of the pH in $H_{2}O$. In the case of the geomorphological features, the DEM derivative *valley depth* is the variable that most significantly impacted on all the predictions. Overall, the use of multispectral bands is more important than the use of DEM derivatives by a ratio of 60–40%. Our study area includes the entire European region, comprising an extensive collection of soil samples with a remarkable diversity and heterogeneity. The analysis presented in this paper provides insights into the potential of machine learning techniques to generalize over a vast geographical area. Nevertheless, given the substantial variations in soil properties across different regions, the validity of these findings for areas beyond Europe should be empirically verified.

The outcomes achieved for some soil variables display more promising results with respect to others; for instance, pH and soil textures (clay, sand, and silt) exhibit superior predictability compared to potassium and nitrogen. Nevertheless, the numerical outcomes, measured in terms of $R^{2}$, closely align with the findings from analogous scientific papers [15], even if on a significantly larger dataset. Our study validates the efficacy of remote sensing methodologies for soil parameter estimation. Despite the presence of estimation errors, these methods offer numerous advantages over conventional approaches. Remote sensing offers significant advantages in terms of spatial coverage, real-time monitoring, non-invasiveness and the ability to capture multispectral information. These advantages make it a powerful tool for soil parameter estimation that complements or even surpasses traditional methodologies in terms of efficiency, accuracy and practicality.

In future works, we plan to include hyperspectral signals in the assessment of the machine learning methods and to compare the quality of the predictions with that of the multispectral signals.

## 6. Code Availability

The code of this paper is available at the GitHub repository specified in (https://github.com/dros1986/soil-characteristics-estimation-from-sentinel-3, accessed on 1 August 2023). It has been developed in Python3. More details on its usage are available on the wiki pages of the repository.

## Figures and Tables

**Table 1:** Structure of the neural network used for the soil variable estimation. *N* represents 21 if only multispectral features are used and 29 if geomorphological features are added. *M* is the number of outputs of the neural network, which can be 1 for the single-variable estimation or 12 for the multi-variable estimation.

Stage	Operation	Output Size
Preprocessing	Input	*N*
Encoding	Linear + Hardswish	32
	Linear + Tanhshrink	128
	Linear + Hardswish	32
	Linear	*M*
	Total parameters	32N+8192+32M

**Table 2:** Variables considered by the state-of-the-art methods composing the state of the art related to the estimation of soil parameters from multispectral signals. Most of these methods consider the SOC as the most important variable. Our work goes further and provides all the necessary tools to estimate almost all soil characteristics simultaneously.

Method	Coarse	Clay	Silt	Sand	pH${}_{CaCl_{2}}$	pH${}_{H_{2}O}$	SOC	CaCO${}_{3}$	N	P	K	CEC	Area
Meng et al. [19]							✓						North east China (315 samples)
Forkuor et al. [13]		✓	✓	✓			✓		✓			✓	Rural watershed (580 km${}^{2}$)
Safanelli et al. [14]		✓		✓		✓	✓	✓				✓	European croplands (7142 samples)
Trontelj et al. [22]									✓	✓	✓		Slovenia (350 samples)
Li et al. [23]		✓					✓		✓				19 sampling sites (180 samples)
Zhou et al. [15]							✓		✓				Switzerland (150 samples)
Our Proposal	✓	✓	✓	✓	✓	✓	✓	✓	✓	✓	✓	✓	Europe (20,000 samples)

**Table 3:** Performance of the considered methods measured in terms of $R^{2}$, RMSE and MAE. For each table, the first group of rows refers to the use of multispectral input (m), while the second group refers to the use of multispectral input and DEM derivatives (m,d). For each input, the underlined values highlight the best methods for each soil variable, while the bold values highlight the best methods on average. For $R^{2}$, the higher the value, the better the method, while for RMSE and MAE, the lower the value, the better the method.

(**a**) $R^{2}$														
**Type**	**Model**	**Silt**	**Sand**	**pH${}_{H_{2}O}$**	**pH${}_{CaCl_{2}}$**	**Coarse**	**Clay**	**P**	**OC**	**N**	**K**	**CaCO** ${}_{3}$	**CEC**	**avg**
(**m**)	**GB**	0.13	0.18	0.42	0.41	0.05	0.18	0.08	0.16	0.11	0.08	0.25	0.08	0.18
	**RF**	0.19	0.25	0.48	0.47	0.06	0.26	0.07	0.13	0.12	0.10	0.33	0.11	0.21
	**SVM**	0.13	0.19	0.44	0.44	0.04	0.14	0.07	0.11	0.07	0.04	0.14	0.02	0.15
	**ANN Single**	0.23	0.28	0.54	0.53	0.11	0.26	0.13	0.20	0.17	0.13	0.37	0.12	0.26
	**ANN Multi**	0.25	0.30	0.51	0.50	0.13	0.26	0.12	0.21	0.15	0.18	0.43	0.14	**0.27**
(m,d)	**GB**	0.22	0.27	0.47	0.47	0.14	0.28	0.13	0.19	0.13	0.10	0.33	0.14	0.24
	**RF**	0.36	0.41	0.54	0.54	0.16	0.39	0.12	0.20	0.17	0.12	0.41	0.22	0.30
	**SVR**	0.27	0.32	0.55	0.54	0.12	0.31	0.14	0.19	0.17	0.10	0.34	0.17	0.27
	**ANN Single**	0.33	0.39	0.57	0.57	0.17	0.35	0.12	0.25	0.20	0.12	0.46	0.20	**0.31**
	**ANN Multi**	0.34	0.39	0.56	0.56	0.14	0.38	0.12	0.25	0.21	0.15	0.44	0.23	**0.31**
(**b**) RMSE														
**Type**	**Model**	**Silt**	**Sand**	**pH${}_{H_{2}O}$**	**pH${}_{CaCl_{2}}$**	**Coarse**	**Clay**	**P**	**OC**	**N**	**K**	**CaCO** ${}_{3}$	**CEC**	**avg**
(m)	**GB**	0.96	0.92	0.76	0.77	0.96	0.89	0.87	0.90	0.95	1.04	0.88	0.99	0.91
	**RF**	0.92	0.88	0.72	0.73	0.95	0.84	0.87	0.91	0.95	1.03	0.83	0.97	0.88
	**SVR**	0.95	0.91	0.75	0.75	0.96	0.91	0.88	0.93	0.97	1.07	0.94	1.02	0.92
	**ANN Single**	0.90	0.86	0.67	0.69	0.91	0.85	0.85	0.88	0.92	1.02	0.80	0.97	**0.86**
	**ANN Multi**	0.88	0.85	0.70	0.71	0.91	0.84	0.86	0.87	0.93	1.00	0.75	0.97	**0.86**
(m,d)	**GB**	0.90	0.86	0.73	0.73	0.91	0.83	0.85	0.88	0.94	1.03	0.83	0.96	0.87
	**RF**	0.82	0.78	0.68	0.68	0.90	0.77	0.85	0.88	0.92	1.02	0.78	0.91	**0.83**
	**SVR**	0.87	0.84	0.67	0.68	0.92	0.82	0.84	0.88	0.92	1.04	0.83	0.94	0.85
	**ANN Single**	0.84	0.79	0.66	0.66	0.90	0.79	0.86	0.86	0.91	1.02	0.75	0.92	**0.83**
	**ANN Multi**	0.85	0.80	0.67	0.67	0.90	0.79	0.86	0.85	0.90	1.03	0.79	0.92	0.84
(**c**) MAE														
**Type**	**Model**	**Silt**	**Sand**	**pH${}_{H_{2}O}$**	**pH${}_{CaCl_{2}}$**	**Coarse**	**Clay**	**P**	**OC**	**N**	**K**	**CaCO** ${}_{3}$	**CEC**	**avg**
(m)	**GB**	0.77	0.76	0.63	0.64	0.72	0.69	0.61	0.58	0.63	0.53	0.50	0.69	0.65
	**RF**	0.73	0.72	0.57	0.58	0.71	0.64	0.61	0.58	0.63	0.52	0.45	0.67	0.62
	**SVR**	0.77	0.75	0.59	0.60	0.67	0.66	0.57	0.52	0.59	0.48	0.42	0.66	0.61
	**ANN Single**	0.70	0.68	0.54	0.54	0.68	0.64	0.57	0.57	0.62	0.51	0.43	0.67	**0.60**
	**ANN Multi**	0.70	0.68	0.56	0.57	0.69	0.65	0.59	0.54	0.61	0.52	0.42	0.66	**0.60**
(m,d)	**GB**	0.73	0.72	0.61	0.61	0.67	0.65	0.59	0.56	0.62	0.52	0.47	0.66	0.62
	**RF**	0.65	0.63	0.54	0.53	0.67	0.58	0.59	0.55	0.60	0.51	0.42	0.61	0.57
	**SVR**	0.69	0.66	0.53	0.53	0.64	0.58	0.53	0.48	0.54	0.46	0.38	0.60	**0.55**
	**ANN Single**	0.66	0.63	0.52	0.52	0.66	0.58	0.59	0.53	0.57	0.52	0.39	0.60	0.56
	**ANN Multi**	0.68	0.65	0.53	0.53	0.67	0.59	0.59	0.53	0.59	0.52	0.44	0.61	0.58

**Table 4:** The best methods for the estimation of each soil parameter, considering both types (m) and (m,d). The methods in bold represent the best type with respect to (m) and (m,d) for each metric and each soil parameter.

Soil	(m)			(m,d)		
**Parameter**	$R^{2}$	**RMSE**	**MAE**	$R^{2}$	**RMSE**	**MAE**
silt	ANN Multi	ANN Single	ANN Multi	**RF**	**RF**	**RF**
sand	ANN Multi	ANN Multi	ANN Multi	**RF**	**RF**	**RF/ANN Single**
pH${}_{H_{2}O}$	ANN Single	ANN Single	ANN Single	**ANN Single**	**ANN Single**	**ANN Single**
pH${}_{CaCl_{2}}$	ANN Single	ANN Single	ANN Single	**ANN Single**	**ANN Single**	**ANN Single**
coarse	ANN Multi	ANN Single/ANN Multi	ANN Multi	**ANN Single**	**RF/ANN Single/ANN Multi**	**SVR**
clay	RF/ANN Single/ANN Multi	RF/ANN Multi	RF/ANN Single/ANN Multi	**RF**	**RF**	**RF/SVR/ANN Single**
P	ANN Single	ANN Single	ANN Single	**SVR**	**SVR**	**SVR**
OC	ANN Multi	ANN Multi	ANN Multi	**ANN Single/ANN Multi**	**ANN Multi**	**SVR**
N	ANN Single	ANN Single	ANN Single	**ANN Multi**	**ANN Multi**	**SVR**
K	**ANN Multi**	**ANN Multi**	ANN Multi	ANN Multi	RF/ANN Single	**SVR**
CaCO_3_	ANN Multi	**ANN Multi**	ANN Multi	**ANN Single**	**ANN Single**	**SVR**
CEC	ANN Multi	RF/ANN Single/ANN multi	ANN Multi	**ANN Multi**	**RF**	**SVR/ANN Single**
avg	ANN Multi	ANN Single/ANN Multi	ANN Multi	**ANN Single/ANN Multi**	**RF/ANN Single**	**SVR**

## Data Availability

Not applicable.

## Abbreviations

The following abbreviations are used in this manuscript:

MDPI	Multidisciplinary Digital Publishing Institute
DOAJ	Directory of Open Access Journals
TLA	Three-letter acronym
LD	Linear dichroism
